# Characterization of differentially expressed and lipid metabolism-related lncRNA-mRNA interaction networks during the growth of liver tissue through rabbit models

**DOI:** 10.3389/fvets.2022.998796

**Published:** 2022-09-01

**Authors:** Guoze Wang, Maolin Li, Yi Wang, Binbin Wang, Hanxu Pu, Jinxin Mao, Shuai Zhang, Shi Zhou, Peng Luo

**Affiliations:** ^1^Key Laboratory of Environmental Pollution Monitoring and Disease Control, Ministry of Education, School of Public Health, Guizhou Medical University, Guiyang, China; ^2^Department of Interventional Radiology, The Affiliated Hospital of Guizhou Medical University, Guiyang, China; ^3^College of Food and Biological Engineering, Chengdu University, Chengdu, China; ^4^College of Pharmaceutical Sciences, Southwest University, Chongqing, China

**Keywords:** lipid metabolism, liver, lncRNA, mRNA, rabbit model

## Abstract

**Background:**

Characterization the long non-coding RNAs (lncRNAs) and their regulated mRNAs involved in lipid metabolism during liver growth and development is of great value for discovering new genomic biomarkers and therapeutic targets for fatty liver and metabolic syndrome.

**Materials and methods:**

Liver samples from sixteen rabbit models during the four growth stages (birth, weaning, sexual maturity, and somatic maturity) were used for RNA-seq and subsequent bioinformatics analyses. Differentially expressed (DE) lncRNAs and mRNAs were screened, and the *cis*/*trans*-regulation target mRNAs of DE lncRNAs were predicted. Then the function enrichment analyses of target mRNAs were performed through Gene ontology (GO) and Kyoto Encyclopedia of Genes and Genomes (KEGG) pathways, respectively. The target protein interaction (PPI) and lncRNA-mRNA co-expression networks were constructed using string version 11.0 platform and R Stats. Finally, six lncRNAs and six mRNAs were verified taking RT-qPCR.

**Results:**

Liver Oil Red O detection found that the liver showed time-dependent accumulation of lipid droplets. 41,095 lncRNAs, 30,744 mRNAs, and amount to 3,384 DE lncRNAs and 2980 DE mRNAs were identified from 16 cDNA sequencing libraries during the growth of liver. 689 out of all DE lncRNAs corresponded to 440 DE mRNAs by cis-regulation and all DE mRNAs could be regulated by DE lncRNAs by trans-regulation. GO enrichment analysis showed significant enrichment of 892 GO terms, such as protein binding, cytosol, extracellular exsome, nucleoplasm, and oxidation-reduction process. Besides, 52 KEGG pathways were significantly enriched, including 11 pathways of lipid metabolism were found, like Arachidonic acid metabolism, *PPAR* signaling pathway and Biosynthesis of unsaturated fatty acids. After the low expression DE mRNAs and lncRNAs were excluded, we further obtained the 54 mRNAs were regulated by 249 lncRNAs. 351 interaction pairs were produced among 38 mRNAs and 215 lncRNAs through the co-expression analysis. The PPI network analysis found that 10 mRNAs such as 3β-Hydroxysteroid-Δ24 Reductase (*DHCR24*), lathosterol 5-desaturase (*SC5D*), and acetyl-CoA synthetase 2 (*ACSS2*) were highly interconnected hub protein-coding genes. Except for *MSTRG.43041.1*, the expression levels of the 11 genes by RT-qPCR were the similar trends to the RNA-seq results.

**Conclusion:**

The study revealed lncRNA-mRNA interation networks that regulate lipid metabolism during liver growth, providing potential research targets for the prophylaxis and treatment of related diseases caused by liver lipid metabolism disorders.

## Background

As the veritable metabolic factory, the liver is the largest solid organ of mammals, which performs important metabolic functions in the pathophysiological process of the body and is responsible for the metabolism of lipids, sugars, protein and vitamins in the body (1–3). Lipid metabolism is a dynamic biological process, including the synthesis, accumulation and distribution of lipid to various specific tissues, which is one of the most important metabolic functions of the liver (4). Liver is susceptible to a variety of pathogenic factors, causing a variety of acute and chronic liver diseases. Dysregulation of lipid homeostasis in liver is strongly related to the hepatic steatosis and metabolic syndrome. For example, dysregulation of lipid metabolism in liver is crucial to the development of non-alcoholic liver disease (NAFLD) and is connected with metabolic syndromes such as obesity and insulin resistance (5–7). Fatty liver disease and its metabolic syndrome have become an important source of global morbidity and mortality, posing a serious threat to global public health and human health (8, 9). But this moment, the pathogenesis of fatty liver disease is still not completely clear. Thus, in-depth analysis of the related regulatory mechanisms of liver lipid metabolism is consequent to prevent and treat fatty liver disease and its metabolic syndrome.

Non-coding RNAs (nRNAs) are crucial regulators for controlling geneexpression and transducing cellular signals (10). As the most abundant part of transcriptional genome, long non-coding RNAs (LncRNAs) indirectly regulate gene expression through transcriptional regulation, post-transcriptional modification and regulation of miRNA activity, thus changing cell physiology and function (11, 12). In the liver, lncRNA can regulate liver growth, development, metabolism, protein decomposition and other important functions (13). It coordinates the formation of lipid, metabolism and transportation of fatty acids, cholesterol and phospholipids, and the formation of high-density lipoprotein (HDL) and low-density lipoprotein (LDL). The expression changes of lncRNAs in liver are associated with many diseases, and dysregulation of lncRNA leads to abnormal lipid metabolism, which leads to the occurrence of diseases such as NAFLD (7). In addition, the expression of lncRNA showed high degree of tissue and cell specificity (4, 14). Because of this specificity, lncRNAs may be more suitable targets for the treatment of related diseases than existing protein-coding genes (15). At the same time, as a functional molecule, LncRNA may be a biomarker for better feedback of disease status (16). Therefore, the regulation of lncRNA expression has huge potential in the gene prevention and treatment of fatty liver disease, and plays important indicative parts in the diagnosis and prognosis of the disease (17). The function of lncRNA is defined by being predicted its interaction with mRNA. Compared with mRNA and miRNA, there is still a big gap in our understanding to the function of lncRNA, and we have little understanding about the regulation effects of most lncRNAs and their functions in development, physiology and diseases (18). Besides, the potential mechanism of lncRNA mediated the biological process of liver lipid metabolism is still unclear. Therefore, studying the interaction between lncRNA and coding genes related to lipid metabolism during the growth and development of liver has great values for identifying new genomic biomarkers and therapeutic targets for metabolic diseases.

Animal model is an important carrier to study the pathogenesis, therapeutic drugs and prevention strategies for diseases. Currently, the model animals major used in studies related to fatty liver disease are mice and rats. Due to the poor sequence homology and conservatism of lncRNA among different species, the molecular and biological effects of lncRNA obtained in mice and rats are difficult to be applied to related studies in humans (4, 19). Therefore, it is necessary to elucidate the functional regulation of lncRNAs in liver lipid metabolism with the help of a variety of model animals. Rabbits are widely used in biomedical field (20). For example, the rabbit model of hereditary hyperlipidemia with familial hypercholesterolemia has made great contributions to elucidate the pathophysiology about human hypercholesterolemia, lipoprotein metabolism and atherosclerosis (21). In addition, there are few studies describing the expression pattern of lncRNAs in liver under physiological state no matter in animal models or human. Therefore, exploring the lncRNAs and mRNAs expression profiles connected with the regulation of lipid metabolism during the growth and development of rabbit liver will further enrich and improve the research vectors and biomarkers of fatty liver disease, and provide more effective therapeutic targets for human fatty liver disease.

Therefore, with the help of RNA-seq, bioinformatics and molecular biology methods, our research used rabbits as animal models to explore the changes of lncRNA and mRNA transcriptomes in rabbits' liver from birth, weaning, sexual maturity, and finally to somatic maturity, and screened, clarified and verified the lncRNA-mRNA interaction networks that may regulate liver lipid metabolism. We finally obtained 38 Differentially expressed (DE) mRNAs as ACSS2 and 215 DE lncRNAs like *MSTRG.30424.1* that may make vital roles in the regulation of lipid metabolism during rabbit liver growth.

## Materials and methods

### Animal model preparation and collection of liver tissue

According to the growth and development cycle of rabbit, 0 (birth), 35 (weaning), 85 (youth) and 120 (adult) days old healthy purebred Hyla rabbits were used in our study. In order to eliminate the interference of diet, after the rabbits were born, the lactation diet of female rabbits kept the same. After being weaned, all male rabbits used were given water and food freely under the same diet (16% protein, 10.8MJ/kg) and feeding environment. In our study, rabbits were euthanized using inhalation anesthesia (isoflurane) and exsanguination. Four rabbits' samples of liver tissue were collected for each stage, snap frozen in liquid nitrogen, and stored at −80°C for subsequent detection.

### Oil Red O staining of liver tissue

Oil Red O staining for frozen sections of liver tissue were performed according to the steps of the reference (22). Lipid droplets of liver section (red in color) were observed and photographed using the OLYMPUS CX31 (OLYMPUS Corp., Japan).

### Preparation and sequencing of rabbit liver tissue samples

Total RNA of liver samples was obtained using Trizol (Invitrogen, Carlsbad, CA, USA), and was quantified using NanoDrop ND-1000. The RNA integrity (RIN number > 7.0) was assessed by Agilent 2100. Then the RNA libraries were constructed and sequenced by LC-BIO Co., Ltd (Hangzhou, China). Briefly, take 5 μg RNA of per sample and remove rRNA using the Ribo-Zero^TM^ rRNA Removal Kit. First-strand and second-strand cDNA was synthesized using Reverse transcriptase, E.coli DNA polymerase I and RNase H, respectively. And then the cDNA libraries were constructed by PCR amplification and purification, and the average insertion of the final libraries was 300 bp (±50 bp). At last, sequencing through Illumina NovaseqTM 6000, and 150bp paired-end reads were generated.

### Transcripts assembly and lncRNA identification

Removing the adapter sequence and low-quality reads from the raw data using Cutadapt (1.9) and FastQC(V0.10.1) (23), the high-quality Valid reads were aligned to the rabbit reference genome OryCun2.0 (http://ftp://ensembl.org/pub/release-102/fasta/oryctolagus_cuniculus/) using histat2 (2.0.4) (24). Assemble the mapped reads of each sample using StringTie (1.3.4) (25) with default parameters and merge to reconstruct all transcriptomes from samples using gffcompare (github.com/gpertea/gffcompare/). And then StringTie was used to perform expression level for mRNAs by calculating FPKM (26). Afterwards, transcripts that overlapped with known mRNAs and shorter than 200 bp were discarded. CPC(0.9-r2) (27) and CNCI(2.0) (28) were used to eliminate all transcripts with CPC score <-1 and CNCI score <0, and remained were regared as lncRNAs.

### Screening of differentially expressed (DE) mRNAs and lncRNAs

Using StringTie calculated expression levels of mRNAs and lncRNAs through their FPKMs. The DE mRNAs and lncRNAs were screened with log2 (fold change) ≥1/ ≤-1 and with *p*-value < 0.05 using R package DeSeq2 (29).

### Prediction and functional analysis about target mRNAs of lncRNAs

To clarify the function of lncRNAs, we predicted the *cis*/*trans-*regulation target mRNAs of the DE lncRNAs. Coding genes in 100-kb upstream and downstream were selected by Python script, and these were considered the potentially *cis*-regulation target mRNAs, while using LncTar predicted the potentially *trans*-regulation target mRNAs. Gene ontology (GO) (30) and Kyoto Encyclopedia of genes and Genomes (KEGG) (31) pathway analyses of the target mRNAs of candidate DE lncRNAs were then performed, respectively.

### Construction of regulation target protein interaction network

To further study the interaction among the selected mRNAs which were related to lipid metabolism, the PPI network of regulation target proteins was constructed using string version 11.0 platform (32). the species (protein species) and the minimum interaction threshold was set as “Oryctolagus cuniculus” (Rabbit) and “medium confidence” 0.4, respectly. And the remaining parameters remained at the default settings. Then, we used the software of Cytoscape (V3.7.2) (33) to study the topological properties of PPI network and draw the diagram.

### Co-expression network analysis of lncRNA-mRNA

In order to investigate the regulatory network in response to lipid metabolism during the liver growth, co-expression network of DE lncRNA-mRNA was created with the help of R package Stats. Use OmicStudio tools (https://www.omicstudio.cn/tool) to select key node DE lncRNA and DE mRNA for visualization, and screen key lncRNAs and mRNAs through this network.

### Validation of DE lncRNAs and mRNAs by RT-qPCR

Primers for the lncRNAs, mRNAs and internal control (GAPDH) (Additional File 1) were designed using Primer-BLAST. Total RNA of sample was converted to cDNA by using a PrimeScript™ RT Reagent Kit (TAKARA, Dalian, China). The reaction mix was comprised of 1 μl template cDNA, 0.5 μl of 10 μM forward and reverse primers, 5 μl SYBR Premix Ex Taq™ II (TAKARA), and 3 μl ddH_2_O at a final volume of 10 μl. Using a Rotor gene 6000 PCR System (QIAGEN, Hiden, Germany), the reactions were proceeded as follows: 95°C for 30 s, 39 cycles of 95°C for 10 s, 60°C for 1 min, 72°C for 1 min, and melting curve analysis was performed from 58°C to 90°C in 1.5°C increments. Using the 2^−ΔΔCt^ method (34) calculated the relative gene expression levels.

### Statistical analysis

In our study, Body weight, Liver weight and RT-qPCR verification results were expressed as mean ± standard deviation (mean ± SD). Data were subjected to analysis of variance using SPSS 26.0 statistical software (SPSS Inc., Chicago, IL, USA). First, the data were tested for a normal distribution and homogeneity of variance. And the differences between the two groups were compared using independent samples *t*-tests. Differences were considered significant at *P* < 0.05.

## Results

### Growth performance and liver lipid deposition during the rabbit growth

**Figure 1:**
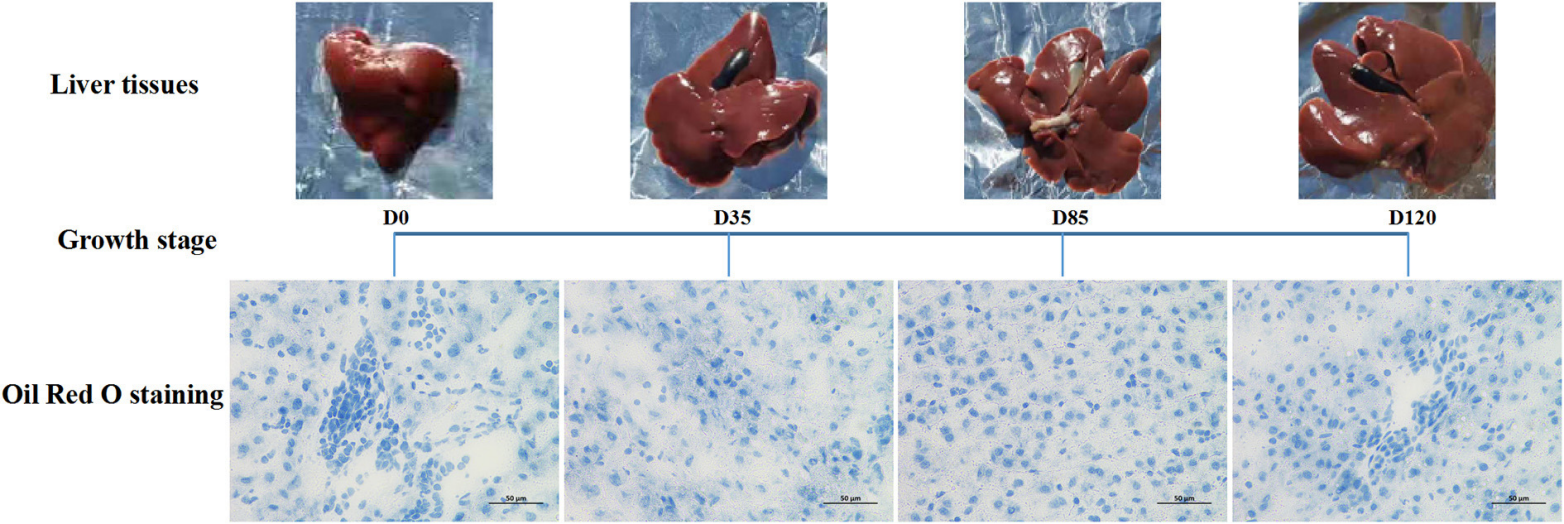
Detection results of Oil Red O in rabbit liver at different growth stages.

The results showed that the body and liver weight of rabbits were increased significantly (*P* < 0.05) with the growth of age (Additional File 2). Liver Oil Red O detection found that the liver showed time-dependent accumulation of lipid droplets, and by day 120, there were obvious large lipid droplets around the liver cells (Figure 1).

### Overview of RNA-seq

We constructed 16 cDNA libraries from the Liver tissues of 0(R0), 35(R35), 85(R85), and 120(R120) days old rabbits. After being sequenced using the Illumina Novaseq^TM^ 6000, and 208.66 Gb raw reads were obtained. Then filtering adapter sequences and low-quality reads, 56,027,094 to 91,373,562 valid reads were obtained from libraries, with the effective ratio and GC content of libraries ranging from 80.82–95.58% and 48.5-52% (Additional File 3). Majority (69.80–77.64%) of valid reads were mapped to the rabbit reference genome, 53.02–60.56% of these had unique genomic positions (Additional File 4). The results showed that the obtained RNA-seq data met the quality control requirements, which could be used for subsequent analysis.

### Identification of lncRNAs and mRNAs in rabbit liver tissue

We obtained 41,095 lncRNAs (Additional File 5A) and 30,744 mRNAs (Additional File 5B) from RNA-seq, and lncRNAs exhibited a higher expression than mRNAs (Additional Figure 1A). All lncRNAs and mRNAs were distributed in 23 chromosomes (Additional Figure 1B). The average length (1579 bp) of the lncRNAs was considerably shorter than the mRNAs (2268 bp) (Additional Figure 1C). Furthermore, the exon number of lncRNAs (average 1) were less than the mRNAs (average 12) (Additional Figure 1D), and the open reading frame (ORF) size in the mRNAs was longer than the lncRNAs (most were within 100 bp) (Additional Figure 1E). In addition, PCA analysis about all of mRNAs and lncRNAs showed that the groups of R0 and R35 had obvious clustering tendency, but samples in groups of R85 and R120 had high similarity.

### Screening of DE lncRNAs and DE mRNAs

**Figure 2:**
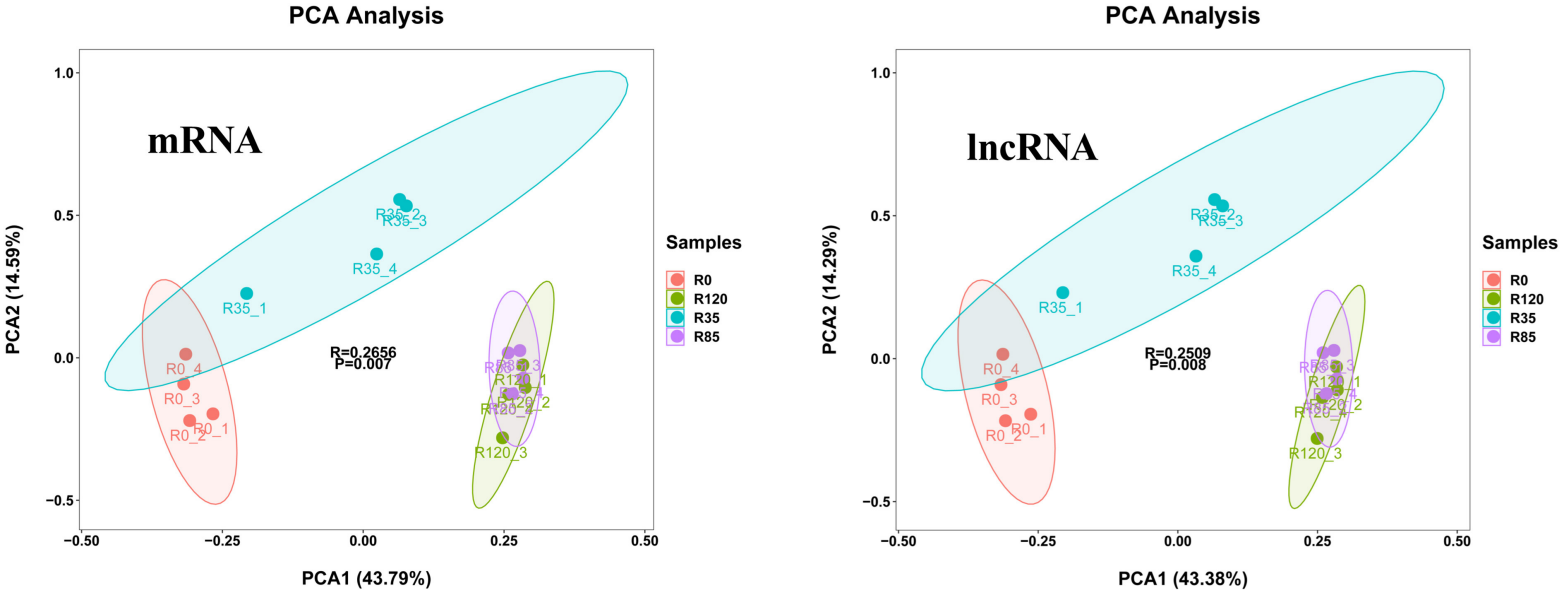
The PCA analysis of DE mRNAs and DE lncRNAs from 16 samples.

**Figure 3:**
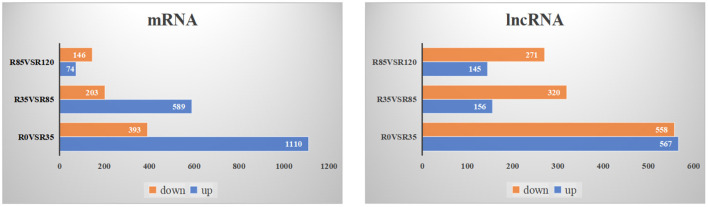
The number of up-regulated and down-regulated DE lncRNAs and DE mRNAs in rabbit liver during the adjacent growth stages.

**Figure 4:**
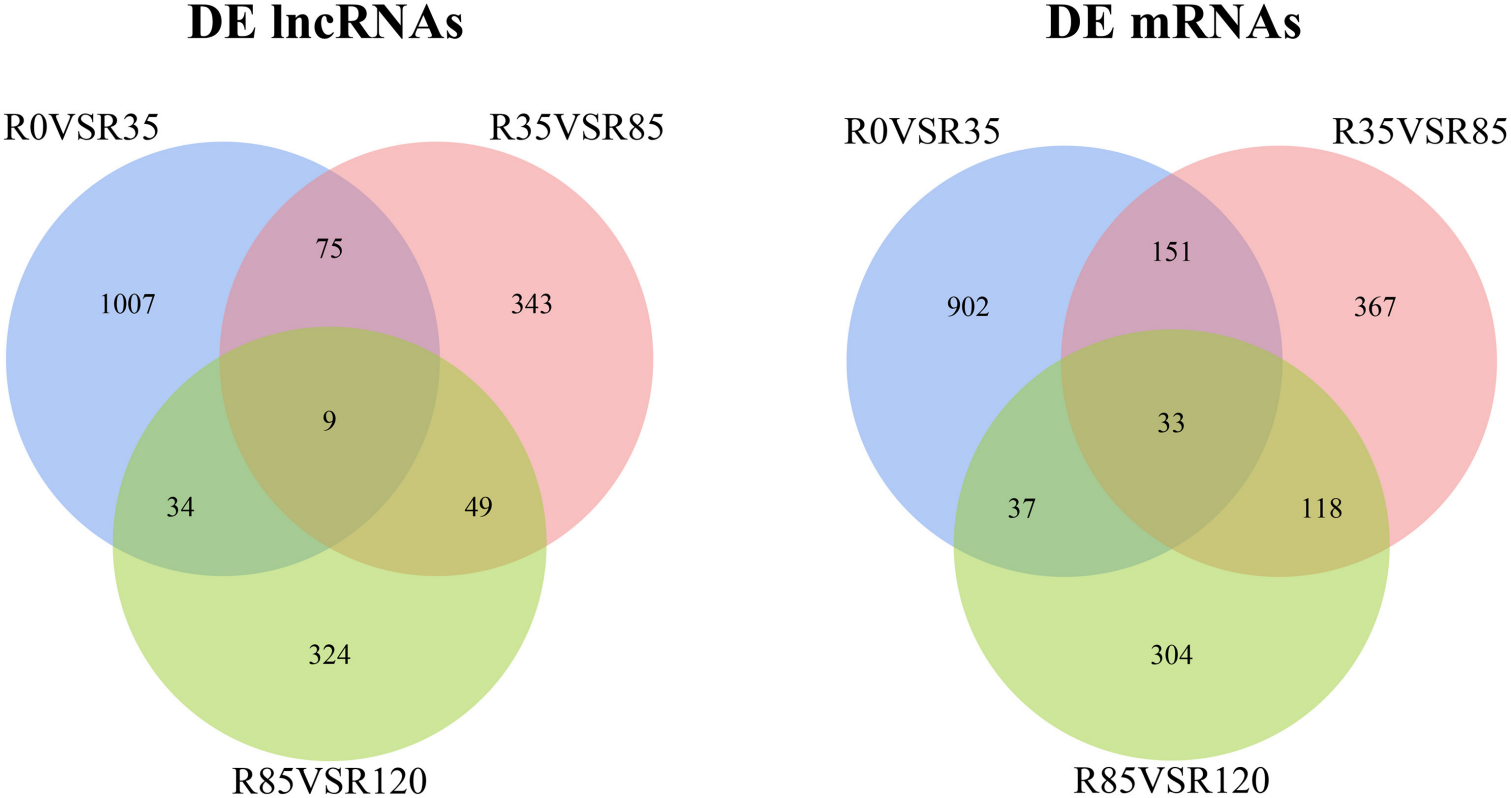
Venn diagrams showing the DE lncRNAs and DE mRNAs in rabbit liver during the growth stages.

The DE lncRNAs and mRNAs were calculated and identified through FPKM using StringTie and R DeSeq2. A total of 3384 DE lncRNAs and 2980 DE mRNAs were screened (*p* < 0.05) during the liver growth when the four growth stages were compared in pairs (Additional File 6). The DE lncRNAs and DE mRNAs across the different libraries were clustered using the PCA analysis (Figure 2), and the clustering results were completely consistent with the clustering trends of total RNAs. When comparing R0 vs R35, R35 vs R85 and R85 vs R120, 1125, 476 and 416 DE lncRNAs and 1503, 792 and 220 DE mRNAs were detected, respectively (Figure 3). From the results, with the growth and development of the liver, the number of DE lncRNAs and mRNAs decreased gradually. Venn diagrams constructed with these DE lncRNAs and mRNAs showed that 9 DE lncRNAs and 33 mRNAs were common existed in four growth stages (Figure 4).

### Functional enrichment network on the target DE mRNAs of DE lncRNAs

**Figure 5:**
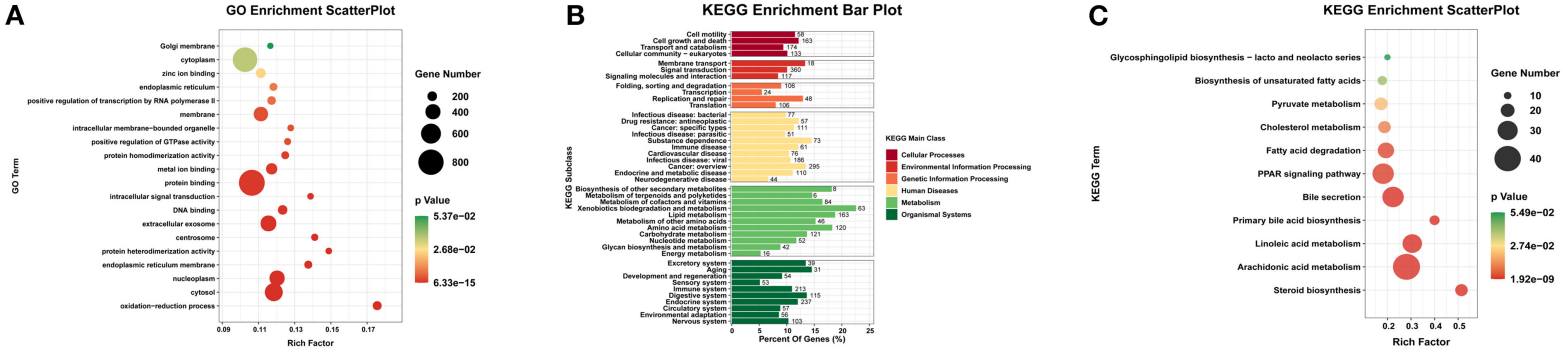
GO and KEGG pathway enrichment analysis of 2980 DE mRNAs. **(A)** The top 20 significant terms of GO enrichment analysis (*p*-value < 0.05). **(B)** Enrichment analysis of KEGG pathway of DE mRNAs. **(C)** The 11 KEGG pathways of lipid metabolism from the total pathways.

The most lncRNAs of rabbit deposited in databases have not yet been functionally annotated. Therefore, prediction of their functions was based on the functions of their *cis*/*trans*-regulated target mRNAs. First, The *cis*-regulated target mRNAs of DE lncRNAs were predicted and 689 out of the 3384 DE lncRNAs corresponded to 440 DE mRNAs. Prediction of the *trans*-regulation showed that all DE mRNAs were regulated by the DE lncRNAs. This indicated that the expression of mRNAs were extremely affected by the change expression of lncRNAs. To better understand the functions of the 2980 DE mRNAs regulated by DE lncRNAs, we performed GO enrichment and KEGG pathway analyses on the mRNAs. GO enrichment analysis showed significant enrichment of 892 GO terms (*P*<0.05), among which, the term of protein binding (GO:0005515) was enriched in the highest with the 821 DE mRNAs. According to the number of DE mRNAs enriched, the significant GO items in the top 20 were displayed, we found the DE lncRNAs affected the growth and development of liver by regulating the expression of mRNAs which play related functions, such as protein binding, cytosol, extracellular exsome, nucleoplasm, and oxidation-reduction process (Figure 5A). In addition, the GO term associated with lipid metabolism like arachidonic acid epoxygenase activity was also significantly enriched. Furthermore, KEGG enrichment analysis was performed. In order to better understand the KEGG pathways about regulated DE mRNAs, we summarized all the pathways predicted into six categories, including Cellular Processes, Genetic Information Processing, Metabolism and Organismal Systems (Figure 5B). These six categories were closely related to the function of the liver, and the results suggested that DE lncRNAs play important roles in the regulation of liver growth and development. Besides, Significant analyses of KEGG pathways found that 52 pathways were significantly enriched. Among these pathways, 11 pathways of lipid metabolism were found, including Arachidonic acid metabolism, Fatty acid degradation, *PPAR* signaling pathway and Biosynthesis of unsaturated fatty acids (Figure 5C). The expression level of mRNAs was also an important factor to determine whether they function or not. To this end, we excluded the low expression DE mRNAs (the mean of total expressions < 10 FPKM) which had been predicted in lipid metabolism, and finally got 54 DE mRNAs that may play roles in lipid metabolism during the growth and development of liver (Additional File 7). After excluding the low-expressed lncRNAs (the mean of total expression < 1 FPKM), we found that the 54 DE mRNAs were *cis* or *trans*-regulated by 249 DE lncRNAs in total (Additional File 8). In addition, through the expression trend analysis, we found that twelve of these mRNAs were consistently up-regulated during all the four growth stages of liver, including *ENSOCUG00000001375, EPHX1, ACSS2, CYP39A1, PCK2* and *APOA5*. While 6 mRNAs, such as *LDHB, FABP7, CA2, ELOVL2, CYP2E1* and *APOA1* were consistently down-regulated.

### Interactions of key lncRNAs and mRNAs involved in liver lipid metabolism of rabbit

**Figure 6:**
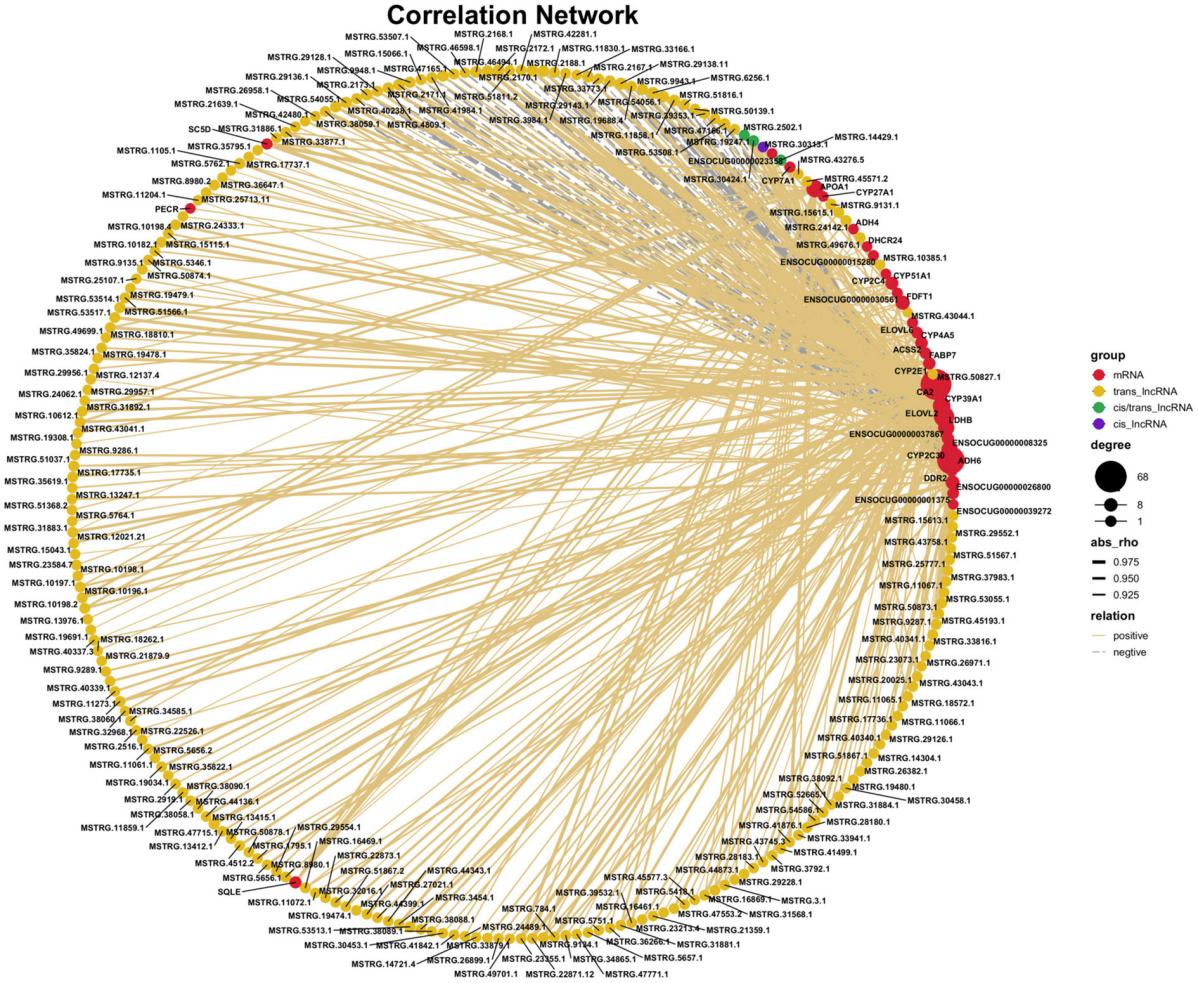
The co-expression analysis of lncRNA-mRNA regulatory network during the growth and development of liver about lipid metabolism in rabbit.

Based on the co-expression theory and the RNAs being selected in the previous step, we constructed the critical lncRNA-mRNA regulatory network during the growth and development of liver about lipid metabolism in rabbit (Figure 6). The results showed that a total of 351 interaction pairs were identified among the 38 DE mRNAs and 215 DE lncRNAs, among which 293 positive interactions and 58 negative interactions were identified, indicating that most of the lncRNAs could positively regulate mRNA expression. Among these lncRNAs, *MSTRG.29138.11* had the most interactions with mRNAs, while the mRNA *CA2* was co-regulated by multiple lncRNAs, and most mRNAs only interacted with a single lncRNA, such as *PECR* and *ADH4*. In addition, the vast majority of lncRNAs interacted with mRNAs through trans-regulated, while only *MSTRG.30313.1* by *cis*-regulated, while *MSTRG.2502.1, MSTRG.30424.1* and *MSTRG.14429.1* by both *cis* and *trans*-regulated. We also found that some unnamed mRNAs, such as *ENSOCUG00000001375, ENSOCUG00000008325*, and *ENSOCUG00000037867* had strong interactions with lncRNAs.

### Construction of target protein-protein interaction (PPI) network

**Figure 7:**
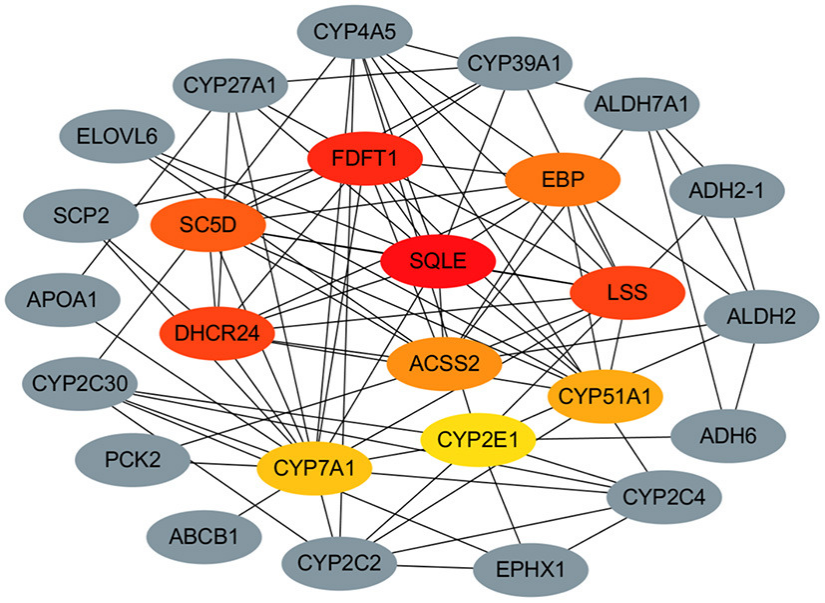
The Protein-protein interaction (PPI) network analysis of the hub regulatory mRNAs in lipid metabolism of liver during the rabbit growth stages.

To gain further insight into lipid metabolism differing between different stages of liver, we performed the PPI network analysis to identify the potential hub regulatory mRNAs. Based on centrality measures and FC values of the 54 DE mRNAs which obtained in the previous step and the String database analysis, the protein-protein interaction network consisting of 26 nodes and 87 edges was constructed using Cytoscape (Figure 7). We identified highly interconnected hub mRNAs of functional network like *SQLE, FDFT1, DHCR24, LSS, SC5D, EBP, ACSS2, CYP51A1, CYP7A1* and *CYP2E1*. Among them, *ACSS2* as the seed mRNA was enriched in the Pyruvate metabolism pathway, while *SQLE, LSS, FDFT1, DHCR24, EBP* and *SC5D* were enriched in the steroid biosynthesis pathway. In addition, *CYP7A1* was enriched in the *PPAR* signaling pathway. Considering their critical positions in the PPI network, these hub mRNAs are expected to be likely more critical than other mRNAs.

### Validation of DE lncRNAs and mRNAs with RT-qPCR

**Figure 8:**
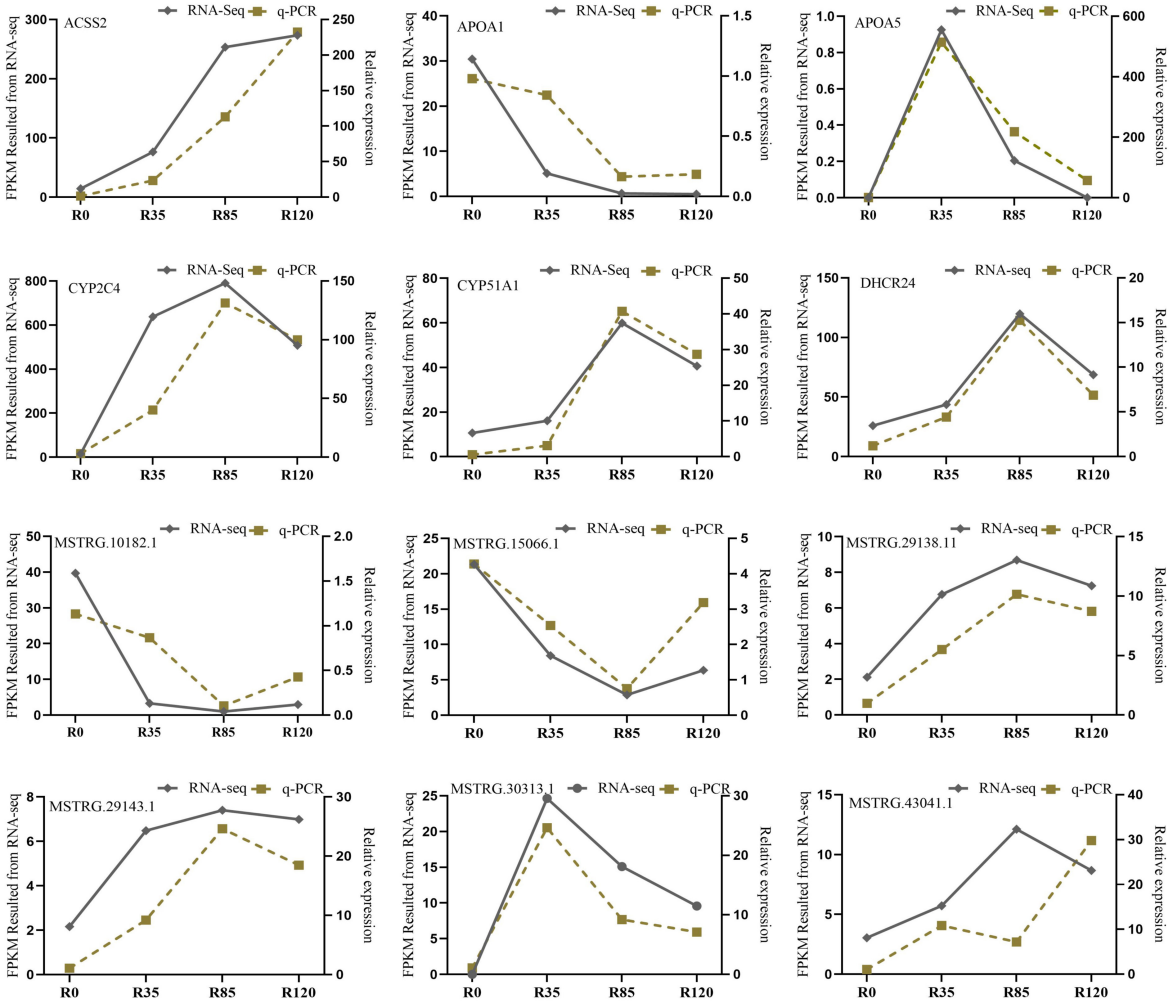
RT-qPCR verification for six lncRNAs and six mRNAs in rabbit four growth stages.

To validate the accuracy of FPKM results from RNA-seq, our study randomly selected six DE lncRNAs and six DE mRNAs to evaluate their expression levels at the four growth stages by RT-qPCR. The RT-qPCR results demonstrated that six lncRNAs (*MSTRG.30313.1, MSTRG.29143.1, MSTRG.29138.11, MSTRG.43041.1, MSTRG.15066.1, MSTRG.10182.1*) and the mRNAs (*APOA5, CYP51A1, DHCR24, CYP2C4, ACSS2, APOA1*) were differentially expressed at the four growth stages. In addition, except for *MSTRG.43041.1*, the expression levels of the rest genes had the similar trends with the RNA-seq results (Figure 8). Therefore, the FPKMs obtained from RNA-seq were accurate and reliable with the detections of RT-qPCR.

## Discussion

Lipid metabolism include a series of biological transformation processes such as the digestion, absorption, translocation, decomposition, transformation, and excretion of lipids in a biological organism (35). It's one of the body's basic metabolic activities and is important for regulation of the body's blood lipid balance and liver fat deposition (36). LncRNA expressions have tissue and disease specificity, because of this property that lncRNAs have been used in clinical study (37). Besides, lncRNAs have been used as biomarkers in a variety of cancers (38, 39). Disorders of liver lipid metabolism can cause non-alcoholic fatty liver disease, non-alcoholic steatohepatitis and even develop liver cancer and liver cirrhosis. However, mild and early fatty liver has no obvious symptoms, so it is not easy to detect. Using the characteristics of DE lncRNAs between normal individuals and those with abnormal hepatic lipid metabolism, early diagnosis, early treatment and prognosis evaluation of fatty liver can be achieved. The study is to explore the expression profiles and interaction networks of lncRNAs and mRNAs related to the regulation of lipid metabolism during the growth and development of liver, and to provide more effective therapeutic targets for human fatty liver diseases.

At present, there were no reports on the physiological changes of liver and transcriptomics research on regulating lipid metabolism in different growth stages of human or common animal models (mice, rats and rabbits). In this study, Oil red O staining detected rabbit liver tissues at different growth stages, and we found that lipid deposition in liver tissues was closely related to animal age. Therefore, it was of great value to explore the transcriptome variation rules of epigenetic modification refracted behind this natural phenomenon and to find the possible regulatory molecular network and major genes. In this study, RNA-seq was performed on 16 liver tissue samples from four important growth stages of rabbits. Through PCA analysis of transcriptomes from different stages samples, we found that the changes of mRNAs and lncRNAs expression in livers of rabbits liver before 35 days were more obvious than those in later stages (85 days and 120 days). The similarity between the 85 days and 120 days was high, and the number of different lncRNAs and mRNAs gradually decreased by comparison in adjacent growth stages. 35 days after birth was the weaning node of rabbits. Before 35 days, rabbits mainly fed on mother's milk. After 35 days, the diets and living environment of experimental rabbits were consistent in each stage. Gene expression is influenced by many factors such as heredity and environment, this shows that age is an important factor for epigenetic changes of liver without changing external diet and environment.

So far, there have been many mechanisms that lncRNAs regulate mRNAs' expression, including chromatin structure modification, RNA transcription, splicing, editing and translation (40), and participate in the occurrence and development of diseases (41). And *cis*-regulation (the expression of its adjacent genes, participating in the regulation of genes in nucleus) and *trans*-regulation (expression of genes across chromosomes, depending on the free energy required for the formation of secondary structure between sequences) are the main ways of lncRNAs regulating mRNAs. Through the prediction of the *cis*/*trans*-regulation of DE mRNAs by DE lncRNAs, we found that all DE mRNAs were regulated by DE lncRNAs in varying degrees, and the regulatory relationship was complex, while the mutual regulation mainly existed in the form of energy combination. Through the functional enrichment analysis of DE mRNA, we found that these DE mRNA could be classified into six categories: Cellular Processes, Genetic Information Processing, Environmental Information Processing, Human Diseases, Metabolism and organic Systems. The functional attributes of genes and their expression level in organs or cells are important factors to determine their function realization (42). When we excluded some genes with low FPKM, we further obtained that 54 DE mRNAs may play the key roles in lipid metabolism during the growth and development of liver, including *EPHX1, ACSS2* and *CYP39A1*, which were continuously up-regulated in four growth stages, and *LDHB, CA2, ELOVL2*, which were continuously down-regulated, and these DE mRNAs were regulated by 249 lncRNAs. To further screen the DE lncRNAs that regulated these 54 mRNAs, the number of DE lncRNAs was reduced from 249 to 215 again by co-expression analysis, and we found that most of lncRNAs had positive regulation with mRNAs. Among 215 lncRNAs, the mRNAs regulated by *MSTRG.29138.1* was the largest, including *LDHB, ELOVL2, ADH6, FDFT1* and so on.

It is of great value to further identify the potential central regulatory mRNAs and clarify the core target of regulating hepatic lipid metabolism for the prevention and treatment of diseases related to hepatic lipid metabolism disorder. With the help of PPI network analysis, we found hub mRNAs with highly interconnected functional networks from 54 DE mRNAs, including *SC5D, EBP, ACSS2* and *CYP7A1*, among which *ACSS2* was the seed mRNA. Related studies have found that *ACSS2* is located in the cytoplasm and nucleus of mammals, and which is the main subtype that catalyzes the production of acetyl coenzyme A from free acetic acid to synthesize fatty acids (43), and lacking *ACSS2* significantly reduced body weight and reversed hepatic steatosis in a diet-induced obesity mice model, suggesting that *ACSS2* may have a therapeutic role in the treatment of fatty liver. Silencing of hepatic ACSS2 potently suppresses the conversion of fructose into hepatic acetyl-CoA and fatty acids (44). In addition, ACSS2 may be an important linker in obesity-related myeloma (45). Cytochrome P450 protein is a monooxygenase that catalyzes many reactions of drug metabolism and the synthesis of cholesterol, steroids and other lipids. *CYP51A1* gene encodes a member of cytochrome P450 superfamily of enzymes which is related to lipid metabolism (46). *CYP51A1* is highly expressed in rabbit liver tissue, and its expression gradually increases with age. In the early growth and development of rabbits, a large amount of synthetic cholesterol is required to participate in the growth and development of the body and cell life activities, such as the formation of cholic acid, the formation of cell membranes and the synthesis of hormones. *CYP51A1* is involved in many reactions in the synthesis of cholesterol, steroids, and is the key enzyme in the synthesis of cholesterol and other substances. Our research shows that lncRNA *MSTRG.30424.1, MSTRG.28180.1, MSTRG.784.1, MSTRG.8980.1* and *MSTRG.22526.1* can positively regulate *ACSS2*, LncRNA *MSTRG.43041.1, MSTRG.32968.1, MSTRG.34585.1* etc. could positively regulate *CYP51A1*. In addition, It was reported that the siRNAs targeting *DHCR24* protect cells from the liposome-induced cell death, probably by reducing production of reactive oxygen species and lowering the cellular cholesterol in the generation (47). Therefore, the function of most hub mRNAs screened by the research has been reported in lipid metabolism, confirming the accuracy of our screening from the side.

In general, this study explored the expression patterns of lncRNA and mRNA transcripts in rabbit livers at different growth stages, and with the help of PPI and other bioinformatics analysis and screening methods, finally confirmed that 215 DE lncRNAs and their regulated 38 mRNAs mediated the role of lipid metabolism during the process of liver growth, which provided important reference targets for further research at the cellular and animal levels, and also provided a theoretical reference for the regulation of clinical intervention targets of liver lipid metabolism disorders.

## Conclusions

In this study, the main key 38 mRNAs affecting liver lipid metabolism were screened in rabbit models at different growth stages, including *ACSS2, CYP51A1* and *DHCR24*, and the potential 215 lncRNAs regulating these key mRNAs were also identified, like *MSTRG.30424.1, MSTRG.8980.1, MSTRG.43041.1, MSTRG.32968.1*. It provides transcriptome data reference for the study and application of rabbit model in liver disease, and also provides potential research targets for the prevention and treatment of related diseases caused by liver lipid metabolism disorder.

## Data availability statement

Most of the tables in this study are provided as the additional files, and the whole RNA-seq reads of 16 rabbit samples can be found at http://www.ncbi.nlm.nih.gov/sra with the accession codes (BioProject ID: PRJNA865562).

## Ethics statement

The animal study was reviewed and approved by Guizhou Medical University experimental animal operation regulations and welfare management committee.

## Author contributions

SZho and PL made the same contribution to the paper. PL and GW conceived and designed the research. GW and ML conducted the experiment and wrote the paper. JM and SZha analyzed the data. YW, BW, and HP modified manuscript. All authors read and approved the final manuscript.

## Funding

This study was supported by China Postdoctoral Foundation (2021M700970), the Guizhou Science Foundation ZK[2021]169, the Foundation of Guizhou Educational Committee (No. KY [2021]008), and the Young Talents Project of Education Department of Guizhou Province KY[2021]155.

## Conflict of interest

The authors declare that the research was conducted in the absence of any commercial or financial relationships that could be construed as a potential conflict of interest.

## Publisher's note

All claims expressed in this article are solely those of the authors and do not necessarily represent those of their affiliated organizations, or those of the publisher, the editors and the reviewers. Any product that may be evaluated in this article, or claim that may be made by its manufacturer, is not guaranteed or endorsed by the publisher.

## Supplementary material

Additional Figure 1 Identification of lncRNA and mRNA expression profiles of liver during the rabbit growth stages. **(A)** Expression level analysis and the total number of lncRNAs and mRNAs. **(B)** The quantitative distribution of lncRNAs and mRNAs in each Chromosome. **(C)** The Length distribution of lncRNAs and mRNAs. **(D)** Exon number distribution of lncRNAs and mRNAs. **(E)** Distribution of open reading frame (ORF) length in lncRNAs and mRNAs.

## Abbreviations

NRNA: noncoding RNA
lncRNA: long noncoding RNA
DE: differentially expressed
GO: gene ontology
KEGG: Kyoto Encyclopedia and Genomes
PPI: protein-protein interaction
DHCR24: 3β-Hydroxysteroid-Δ24 Reductase
SC5D: lathosterol 5-desaturase
ACSS2: acetyl-CoA synthetase 2
NAFLD: non-alcoholic liver disease
HDL: high-density lipoprotein
LDL: low-density lipoprotein.
