# Supplementary figures and images for: Characterization of differentially expressed and lipid metabolism-related lncRNA-mRNA interaction networks during the growth of liver tissue through rabbit models
Source: Front Vet Sci. 2022 Sep 1;9:998796. doi: 10.3389/fvets.2022.998796 (PMC9477072; doi:10.3389/fvets.2022.998796)

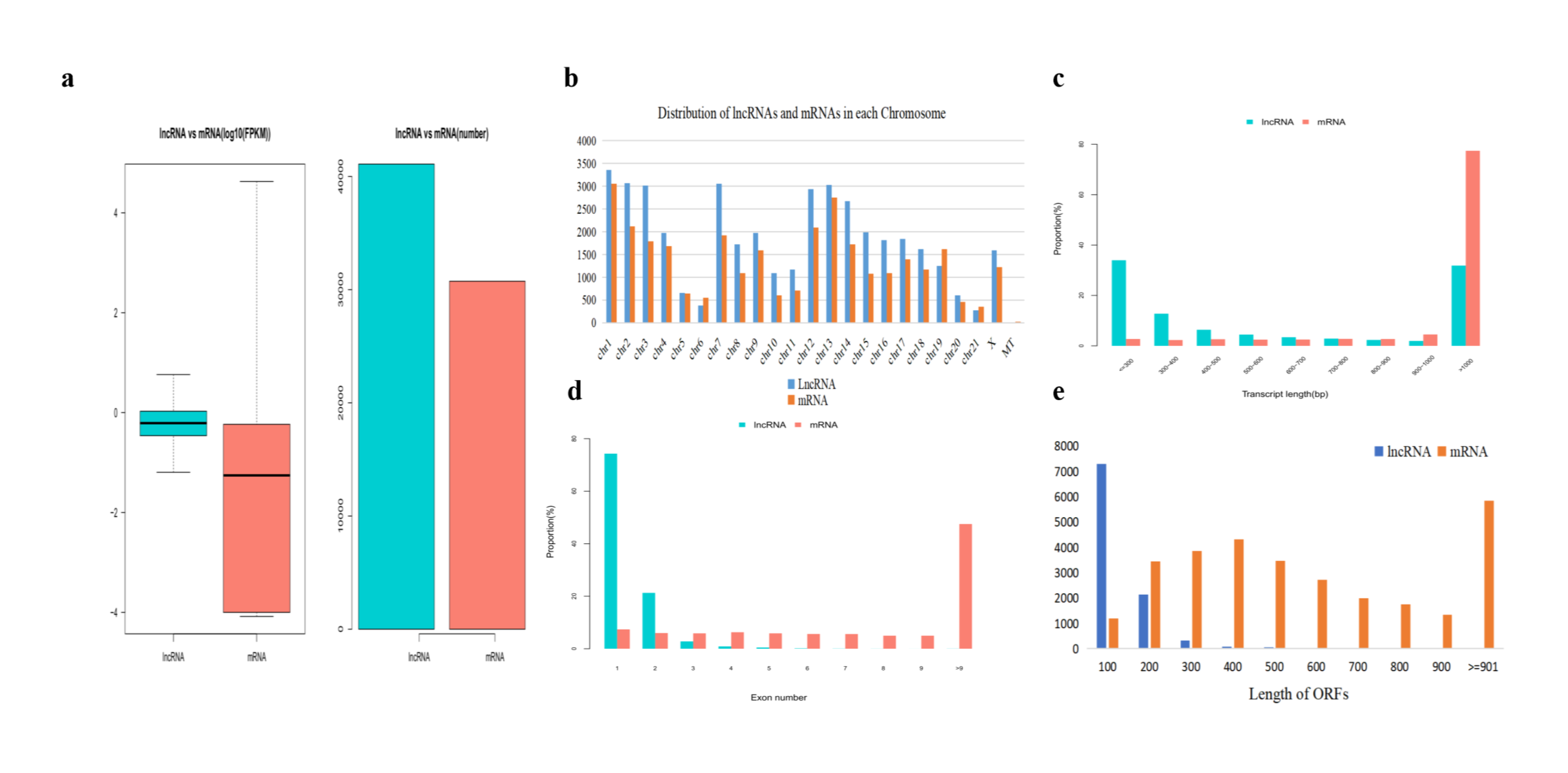

Supplement: Additional Figure 1 — Identification of lncRNA and mRNA expression profiles of liver during the rabbit growth stages. (A) Expression level analysis and the total number of lncRNAs and mRNAs. (B) The quantitative distribution of lncRNAs and mRNAs in each Chromosome. (C) The Length distribution of lncRNAs and mRNAs. (D) Exon number distribution of lncRNAs and mRNAs. (E) Distribution of open reading frame (ORF) length in lncRNAs and mRNAs. [file Data_Sheet_1.ZIP › Additional files/ Additional Fig.1.tif]
